# Rheology and Microstructures of Rennet Gels from Differently Heated Goat Milk

**DOI:** 10.3390/foods9030283

**Published:** 2020-03-04

**Authors:** Zorana Miloradovic, Nemanja Kljajevic, Jelena Miocinovic, Steva Levic, Vladimir B. Pavlovic, Marijana Blažić, Predrag Pudja

**Affiliations:** 1 Department for Animal Source Food Technology, Faculty of Agriculture, University of Belgrade, Nemanjina 6, 11081 Belgrade, Serbia; nemanja.kljajevic@agrif.bg.ac.rs (N.K.); jmiocin@agrif.bg.ac.rs (J.M.); pudja@agrif.bg.ac.rs (P.P.); 2 Department for Food Technology and Biochemistry, Faculty of Agriculture, University of Belgrade, Nemanjina 6, 11081 Belgrade, Serbia; stevalevic@gmail.com; 3 Department for Mathematics and Physics, Faculty of Agriculture, University of Belgrade, Nemanjina 6, 11081 Belgrade, Serbia; vlaver@agrif.bg.ac.rs; 4 Institute of Technical Sciences of Serbian Academy of Sciences and Arts, Knez Mihailova 35/IV, 11000 Belgrade, Serbia; 5 Department of Food Technology, Karlovac University of Applied Sciences, Trg J.J. Strossmayera 9, 47000 Karlovac, Croatia; marijana.blazic@vuka.hr

**Keywords:** goat milk, rheology, microstructure, heat treatment, curd yield

## Abstract

Rennet coagulation of goat milk heated to 65 °C/30 min (Gc), 80 °C/5 min (G8) and 90 °C/5 min (G9) was studied. A rheometer equipped with a vane geometry tool was used to measure milk coagulation parameters and viscoelastic properties of rennet gels. Yield parameters: curd yield, laboratory curd yield and curd yield efficiency were measured and calculated. Scanning electron microscopy of rennet gels was conducted. Storage moduli (G’) of gels at the moment of cutting were 19.9 ± 1.71 Pa (Gc), 11.9 ± 1.96 Pa (G8) and 7.3 ± 1.46 Pa (G9). Aggregation rate and curd firmness decreased with the increase of milk heating temperature, while coagulation time did not change significantly. High heat treatment of goat milk had a significant effect on both laboratory curd yield and curd yield. However, laboratory curd yield (27.7 ± 1.84%) of the G9 treatment was unreasonably high compared to curd yield (15.4 ± 0.60%). The microstructure of G9 was notably different compared to Gc and G8, with a denser and more compact microstructure, smaller paracasein micelles and void spaces in a form of cracks indicating weaker cross links. The findings of this study might serve as the bases for the development of different cheese types produced from high-heat-treated goat milk.

## 1. Introduction

Rennet coagulation is a fundamental part of the cheese making process, affecting both the quality and yield of cheese [1]. Factors affecting rennet coagulation of cow milk have been studied continually for decades and continue to be of interest [2,3]. Scientific research into the rennet coagulation of goat milk is much less common but is increasing due to the rapid increase in goat milk and cheese production over the last two decades [4,5]. 

Rheological measurements are a good tool for investigating rennet coagulation and the performances of different milk types. The viscoelastic nature of rennet gels is determined by rheological parameters such as storage modulus (G’) and loss modulus (G’’) [3]. These parameters are often used to calculate milk coagulation properties (MCP) described by the following parameters: coagulation time (CT), aggregation rate (AR) and curd firmness (CF) [4,6]. The rheological behavior of milk upon coagulation could be influenced by numerous factors regarding processing conditions and raw material quality, and it is directly related to the quality of the resulting cheese. Therefore, analyzing all the mentioned rheological parameters is important for the evaluations of processes and equipment, for process control and product handling and for characterization of food products for consumer acceptability [7].

The most important factor affecting cheese yield is milk composition (in particular, the concentrations of fat and protein) influenced by the season, but also by the udder health in goats [8]. In order to enhance the yield, cheese makers commonly standardize milk fat and protein content by adding different kinds of protein powders [9]. However, there are only a few companies in Europe and not a single one in Serbia producing goat milk protein powders. Bearing in mind that goat cheeses are mainly manufactured by small artisan cheese-makers [10,11], the usage of imported protein powders for cheese yield enhancement would not be a cost-effective option.

The application of high-heat treatment to cheese milk is one option for cheese yield enhancement. However, when this method is applied to cow milk, it leads to diminished renneting properties, which makes it unacceptable [12]. On the other hand, the heating of goat milk to a high temperature has less pronounced effects on rennet coagulation parameters compared to cow milk [13,14,15], and hence could be successfully applied in cheese production. We confirmed this statement in our former study [16], which showed that high pasteurization regimes improve sensorial quality and yield of white brined cheese, and affects its composition, texture and proteolytic changes during ripening.

The influences of different heat treatments on MCP have been studied by a very limited number of authors [15,17,18], and the conclusions are not consistent, particularly where CT is concerned. 

The first objective of this study was to investigate how high pasteurization (80 °C for 5 min and 90 °C for 5 min) affects the rennet coagulation properties of goat milk in terms of rheological parameters and microstructure. Secondly, the aim was to investigate the effectiveness of the milk heating regime with regard to the enhancement of curd yield, and how is it correlated with MCP in terms of CT, AR and CF. The microstructure of gels is a consequence of the rheological activity of milk and has a direct influence on the texture of final products [19]. Due to that, we conducted the scanning electron microscopy of rennet gels, in order to give deeper insight into the differences caused by heat treatments of goat milk. 

## 2. Materials and Methods

### 2.1. Cheese Manufacture and Curd Yield (CY) Measurement 

The raw milk used in this study (total protein: 2.57 ± 0.10%; fat: 2.75 ± 0.15%; total solids: 10.30 ± 0.04%) was taken from a commercial flock of Saanen goats during the months of June and July. Cheese manufacture was described in detail in our previous study [16]. In summary, a batch of raw milk (60 L) was divided into three equal lots and heated in the cheese vat to 65 °C for 30 min (Gc), 80 °C for 5 min (G8) and 90 °C for 5 min (G9). Using the same procedure, soft cheeses were produced from each lot: at 31 °C, starter culture (0.005% (*w*/*v*), Lyofast MO 030, Clerici-Sacco Group, Cadorago, Italy), CaCl2 (0.02% *w*/*v*) and rennet (0.2 g per 10 L of milk, Caglificio Clerici, Clerici-Sacco Group, Cadorago, Italy) were added. After 45 min of coagulation, the curd was gently cut with a knife into 5 cm cubes, left to rest for 15 min and then transferred to a mold. After draining and pressing, the curd was weighed, and curd yield (CY) was calculated as the weight percentage of curd compared to the weight of the cheese milk used. Each cheese making trial was repeated three times over three consecutive weeks.

### 2.2. Laboratory Curd Yield (LCY) and Curd Yield Efficiency (CYE) 

Laboratory curd yield represents the yield potential of the heated cheese milk. It was measured and calculated according to the slightly modified method of Hallen [20]. From each cheese vat, after the addition of rennet, and gentle stirring for 1 min, 3 × 10 mL aliquots of renneted milk were transferred to conical polypropylene sealed tubes. The tubes were placed in a water bath, at 31 °C. After 30 min, the rennet gel was cut vertically in the shape of a cross with a spatula and then left to rest for an additional 30 min. All samples were then removed from the water bath and centrifuged at room temperature, at 1260 g/25 min in the laboratory centrifuge (Eppendorf AG 5430, Hamburg, Germany). The expelled whey was decanted and the gel was weighed. In order to be comparable with CY, LCY was also expressed as the weight percentage of curd compared to the weight of cheese milk used for the experiment.

In order to calculate CYE [21], CY was compared with LCY (curd yield as percentage of laboratory curd yield—(CY/LCY) × 100). 

### 2.3. Rheological Measurements

At the same time that renneted milk was taken from the cheese vat for the LCY measurements, other 50 mL aliquots were taken from each vat (one per each cheese making trial) for rheological measurements that were conducted with a Kinexus Pro rheometer (Malvern Instruments Ltd., Worcester, UK), equipped with a vane geometry tool. During 1 h of the rennet coagulation, G’, G” and δ were continuously recorded every 5 s, at 1 Hz frequency and 0.01 strain amplitude. Changes in G’ as a function of time were reported. 

Subsequently, a frequency sweep test (0.02–20 Hz) was performed at a constant strain amplitude of 0.01. Changes in G’ and loss tangent (tg δ) as a function of frequency (0–1 Hz) were reported.

From the parameters recorded during the two aforementioned tests, coagulation parameters—AR, CT and CF—were calculated in Origin Pro 8.0 (Origin Lab Corporation, Northampton, MA, USA) as described previously [4,6,22]. 

### 2.4. Scanning Electronic Microscopy (SEM) 

Samples of rennet gel obtained from differently heated goat milk (Gc, G8 and G9) were taken from the interior of a cheese vat 45 min after the addition of rennet. For each cheese making trial, three samples were taken. They were cut into equal particles (≈5 × 5 × 15 mm). Samples were defatted and fixed the same as in the study of Kuo and Gunasekaran [23], and critical point dried with liquid carbon dioxide, using a K850 Critical Point Drier (Quorum Technologies, Laughton, UK).

Dried fractured rennet gel samples were attached to stubs and sputter coated with gold (50 nm) for 100 s at 30 mA (Sputter Coater BAL-TEC SCD 005, Scotia, New York, NY, USA). Samples were examined with a JEOL JSM 6390LV scanning electronic microscope (JEOL, Tokyo, Japan). The SEM operated at 25 kV with a magnification of 5000. 

### 2.5. Statistical Analysis

A one-way ANOVA general linear model (GLM) was used to analyze the effects of heat treatment (as a factor) on milk coagulation parameters (AR, CT and CF) and yield parameters (CY, LCY and CYE).

If the effect was significant, means were compared by least significant difference test (LSD test), with the level of significance at *p* < 0.05. Correlation analysis between coagulation parameters and curd yield was also conducted. 

Calculations were done by Statistica 10.0 software (Stat Sof. Inc., Tulsa, OK, USA).

## 3. Results and Discussion

### 3.1. Rheological Measurements

**Figure 1:**
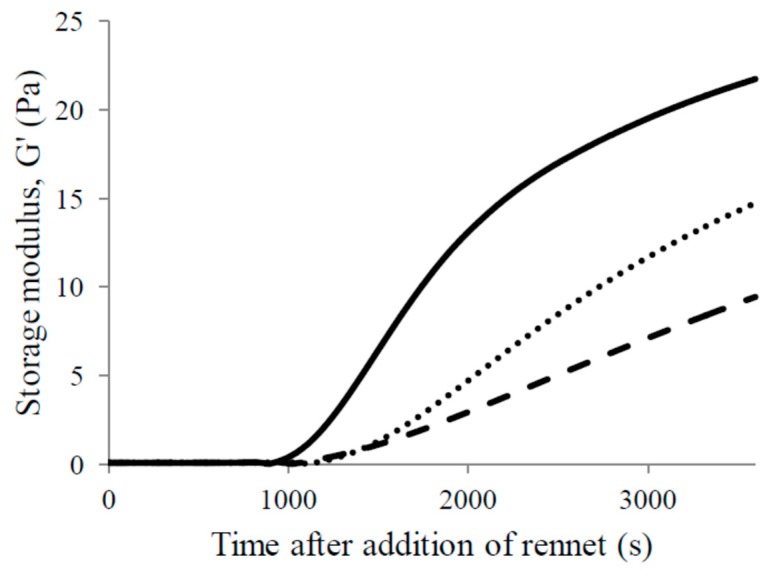
Storage modulus (G’) as a function of time during rennet coagulation of goat milk heated to 65 °C/30 min (──), 80 °C/5 min (⋯⋯) and 90 °C/5 min (**– –**).

Changes of G’ as a function of time are shown in Figure 1. 

The shapes of the three presented curves were notably different. Gel from commonly pasteurized goat milk exhibited a faster increase of G’ illustrating the development of a stronger protein matrix compared to the other two. At the end of the monitored time, the rate of G’ increase slowed down. On the contrary, the gels from highly pasteurized milk, especially G9, showed a slow but continuous increase of G’. At a given time, G’ values of formed gels decreased as the temperature of heat treatment increased.

The proper time for a gel to be cut is an intriguing topic both for cheese makers and scientists. 

While on the one hand it is claimed that it could be very precisely determined empirically by an operator, on the other hand, instrumental techniques are being developed in order to predict the cutting time by determining the exact stiffness of gel at the moment of cutting [24]. Based on the rheolological measurements, Guinee, et al. [25] suggested the fixed storage modulus G’ = 16 Pa as being the proper cutting time stiffness of gel. According to Salvador, et al. [26], the value of the cutting time gel stiffness is G’ = 30 Pa. Recently, Panthi, Kelly, Sheehan, Bulbul, Vollmer and McMahon [3] established the term cutting window as the time in which the value of G’ lies between 35 and 70 Pa, when the gel is ready to be cut. To the authors’ knowledge, to date, no study has been conducted on the determination of the cutting time of gels from high-heat-treated goat milk. Therefore, in the present experiment, cutting time (45 min after the addition of rennet) was determined empirically by cheese makers as described by Castillo, et al. [27]. At that moment, all three gels were visually ready to be cut, although the rheological measurements showed significantly different G’ values: 19.9 ± 1.71 Pa (Gc), 11.9 ± 1.96 Pa (G8) and 7.3 ± 1.46 Pa (G9). Taking into account the different rheometers used in the mentioned studies, the storage moduli of Gc were comparable to the values reported for pasteurized cow milk by Guinee, Pudja and Mulholland [25] and Yu, et al. [28], but lower than the ones reported by Salvador, Arango and Castillo [26] and Panthi, Kelly, Sheehan, Bulbul, Vollmer and McMahon [3]. However, the other two G’ values (for G8 and G9) were much higher than the G’ of cow milk heated to 80 °C/20 min [28] indicating the different effects of high temperature on two milk types. Further investigations, employing some instrumental techniques, for example, light scattering techniques or fluorescence spectroscopy [24], are needed in order to accurately predict the cutting time (or cutting window) for highly pasteurized goat milk. 

**Figure 2:**
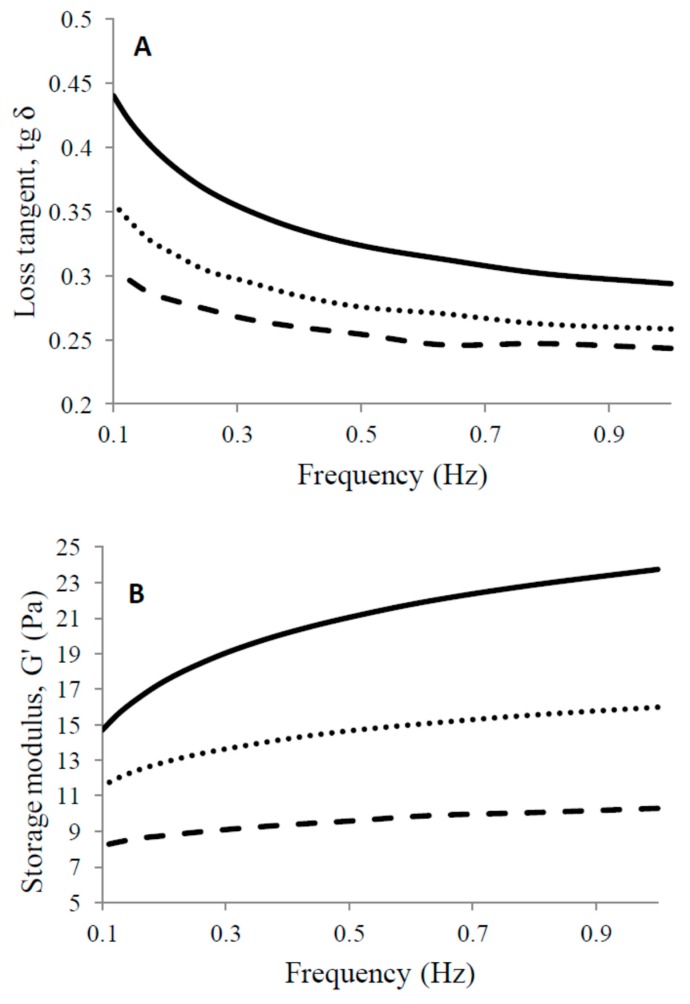
Storage moduli (G’) (**A**) and tg (δ) (**B**) of rennet gels from milk heated to 65 °C/30 min (──), 80 °C/5 min (⋯⋯) and 90 °C/5 min (**– –**) as a function of frequency.

Loss tangent (tg δ) as a function of frequencies (Figure 2A) is a measure of how prone gel is to breakage of protein-protein bonds during the gel rearrangement [3]. 

At low frequencies, a high tg δ indicates that the protein network is more susceptible to rearrangements [29]. The increase of the temperature of goat milk heat treatment was followed by the decrease in tg δ (Figure 2A). Such a result is in accordance with the curd yield measurements (presented later), showing that high-heat treatment favors the retention of whey in the curd. Loss tangent increased with decreasing frequencies for all three heat treatments, which is similar to the performance of cow milk [29]. 

Storage modulus (G’) as a function of frequencies (Figure 2B) measures the strength and number of cross-links in the protein matrix of gel [29]. With an increase of milk heat treatment, G’ decreased at any recorded frequency, again suggesting that the higher the heat treatment of goat milk, the weaker the bonds and the less elastic the nature of gel. 

### 3.2. Coagulation Parameters

**Figure 3:**
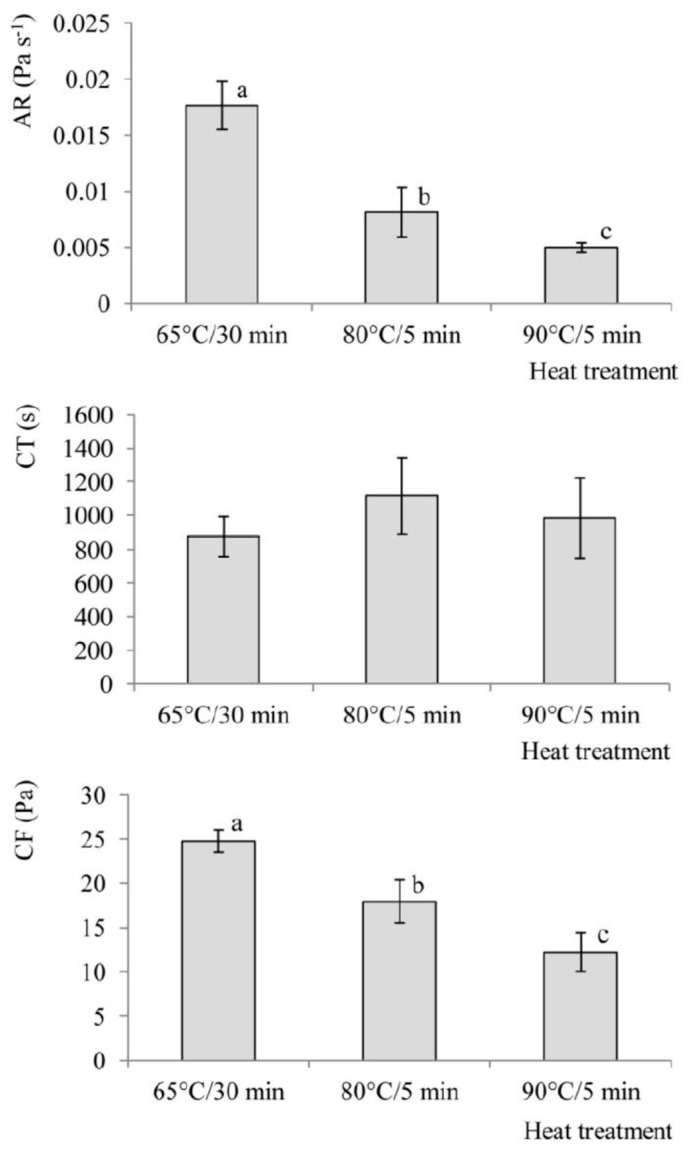
Rennet coagulation parameters: aggregation rate (AR), coagulation time (CT) and curd firmness (CF) of rennet gels from goat milk heated to 65 °C/30 min, 80 °C/5 min and 90 °C/5 min. (a,b,c) Values assigned with different letters differ significantly from one another (*p* < 0.05); results in the graphs are the mean values of three replicates; error bars indicate standard deviations).

The effects of heat treatment on coagulation parameters AR, CT and CF are shown in Figure 3. The results indicate that heat treatment had a significant effect on AR and CF. As the severity of heat treatment increased, the rate of aggregation decreased, and the gel had lower firmness. Similar findings were already reported both for cow and goat milk [18,30,31]. However, the effect of heat treatment on CT was not statistically significant. Literature data on this particular matter vary considerably. Certain authors report that heat treatment of goat milk (70 °C/30 min) does not affect CT significantly [17,18]. Others report that after heating the goat milk to 80 °C/1 min, a 1.3 fold increase of CT occurs, while after 90 °C/1 min it decreases almost to the value measured for raw milk [15]. A group of authors also bring the following results: heating of goat milk to 80 °C/1, 3 or 10 min, does not have a significant effect on CT, while after treatment to 95 °C/1, 3 or 10 min, CT decreases significantly [17]. After summarizing the literature data, and also based on the results of the present study, we hypothesize that in the case of goat milk, there is a temperature interval between 80 °C and 90 °C where heat treatment causes an increase in coagulation time, and that there is also a point above 90 °C where heat treatment causes its decrease. The distinctive performance of goat milk compared to cow milk is explained in the literature by the specific mineral and protein composition of goat milk [32]. 

For heating regimes including the temperature of 90 °C and above, it appears that heat treatment has opposite effects on the coagulation times of goat and cow milks. While it extends CT, and in some cases even prevents the coagulation of cow milk, it does not affect or reduces CT of goat milk. It is mostly agreed in the literature that the second aggregation phase of rennet coagulation is affected by heat treatment [15,33]. The difference in mineral composition that is crucial for the rennet coagulation process, is such that the level of ionic calcium is higher in goat milk [34]. As a result of heating, a certain amount of ionic calcium (Ca^2+^) irreversibly precipitates [15]. Decreased levels of Ca^2+^ imply longer coagulation time for cow milk [34,35]. It could be hypothesized that in the case of the goat milk and heating regimes applied in this study, higher initial levels of Ca^2+^ compared to cow milk appeared to be sufficient for the undisturbed rennet coagulation to occur, even after a certain amount of it precipitated during heating. By engaging the method for quantification of soluble and micellar calcium fractions [36], calcium distribution in goat milk after different heat treatments could be conducted, and the proposed hypothesis could be tested.

Although the basic compositions of cow and goat milk are similar [37], their protein compositions are quite different, which is also mentioned in the literature as being a reason for the different performances of two milk types. The significantly higher amount of β-casein in goat milk compared to cow milk, and a lower ratio of β-lactoglobulin/α-lactalbumine [14], surely change the way that these two milk types coagulate by rennet. But what we consider to be much more significant are the differences between the structures of the casein micelles of these two milk types, especially the different way they transform after heating. 

Firstly, the diameter of cow milk casein micelle does not change significantly after heating, while the goat milk micelle diameter increases by up to 25% [15]. Therefore, in case of goat milk, the collision distance between the micelles decreases, which positively affects the coagulation process [38]. 

Furthermore, it is known that both in goat and cow milks, whey protein/casein (WP/CN) complexes formed after heating create unstable hydrophobic areas on the surfaces of casein micelles. Therefore, heated casein micelles of both goat and cow milks have an increased surface hydrophobicity compared to the unheated ones [39]. Moreover, β-casein, the most hydrophobic of all casein fractions, partly covers the surface of the goat milk micelle [40,41]. Considering the surface hydrophobicity approach [38], more compact cow micelles, whether before or after heating, tend to bury their hydrophobic sites in the interior part. It was stated recently that formation of hydrophobic bonds represents one of the main attractive forces that occurs during renneting [38]. Therefore, after milk heating to 90 °C and above, the initially higher hydrophobicity of the goat milk casein micelle surface further increases. As a result, it becomes more susceptible to aggregation than it was before heating, and also more susceptible than its cow milk counterpart.

But what could be assumed to be the crucial cause for the contrasting performances of heated goat and cow milk with regard to rennet coagulation, are the findings described by Pesic, Barac, Stanojevic, Ristic, Macej and Vrvic [41]. In the model they have proposed, after heat treatment of goat milk (90 °C/10 min) WP/CN complexes are completely micelle-bonded and evenly distributed on the surface of the casein micelle. It has been known for decades that after heat treatment of cow milk, WP/CN complexes can be found both on the surface of the casein micelle and in the serum phase. Accepting that the average size of cow casein micelles is ≈200 nm [42] and the average size of WP/CN complexes is ≈100 nm [39], there is no doubt that by steric hindrance, complexes prevent cow milk micelles from fusion. In other words, by heating, cow milk casein micelles become more stable entities [33]. But if, according to the model of Pesic, Barac, Stanojevic, Ristic, Macej and Vrvic [41], WP/CN complexes do not stand in the way of fusion of goat casein micelles, transformed after heating to 90 °C and above, it is highly likely that, larger and more hydrophobic as they become after heating, they would aggregate more readily.

Since to the author’s knowledge there are no studies dealing with the 80 °C/5 min treatment of goat milk in terms of distribution of aggregates between micelle and serum, we can only hypothesize that unlike the heat treatments at 90 °C, a certain number of serum aggregates do exist, resulting in the increased coagulation time reported by some authors. Further investigations are needed to confirm this hypothesis. 

### 3.3. Curd Yield Parameters 

The effect of heat treatment on laboratory curd yield (LCY), and also on actual curd yield (CY) calculated after the production of cheese curd, is presented in Table 1, along with the curd yield efficiency (CYE). 

As can be seen, heat treatment had a significant effect on all three parameters. When milk was heated to 80 °C/5 min, both laboratory and actual curd yields increased significantly compared to common pasteurization (65 °C/30 min). The yield efficiency did not change significantly. However, after the 90 °C/5 min treatment, compared to the 80 °C/5 min treatment, LCY increased significantly, but the increase of CY was not statistically significant, and hence the CYE appeared significantly lower for this heating regime. This suggests that the increase in yield potential of milk (LCY), caused by the most severe heat treatment applied, could not be fulfilled in a real system.

### 3.4. Correlations between Curd Yield and Coagulation Parameters

Regardless of the numerous studies, the relationship between coagulation parameters and CY is still controversial [43]. It was reported earlier that CF is the primary coagulation parameter that influences cheese yield, and hence economic returns. In general, firm curd improves cheese yield by encouraging retention of milk components [44]. In the present study, however, the results are quite the opposite: the significant decrease of CF caused by high-heat treatment was followed by the increase of curd yield. The reason for the curd yield increase is not correlated to CF; it is caused by better retention of serum, and proteins and fat in the curd from heat treated milk [16].

It may seem reasonable to hold that manipulation with curd of such low firmness would induce the creation of cheese fines and certainly cause some losses of milk components (mainly casein) in whey. Nevertheless, according to our former study [45] the protein composition of whey did not reveal higher casein losses when high-heating regimes (C8 and C9) were undertaken, in comparison with common pasteurization. 

Raynal and Remeuf [15] have concluded that curd shrinkage and the number of reticulation bonds in the rennet gel are not affected by the heating of goat milk, and it is only the gel permeability decrease that causes the loss of whey draining ability. The results of our study confirm this theory: during the 25 min centrifugation (LCY measurement), low gel permeability prevented the whey from being driven out of the gel, but when 2 h of curd pressing was conducted (CY measurement), the protein matrix had enough time to reorganize, shrink and expel a much greater amount of whey. As a result, LCY was unreasonably high in comparison with CY. Being less prone to rearranging, curd from high intensity heat treated goat milk needs more time to expel the whey than curd from commonly pasteurized milk. Our results are in accordance with the conclusions of Nguyen, et al. [46], stating that the presence of disulfide bonds impedes the rearranging ability of proteins in the gels. 

According to statistical analysis, the only significant correlation was found between AR and CY (*p* < 0.05, R = −0.999). The lower aggregation rate contributed to less permeable gel being formed, which resulted in a better incorporation of milk components and serum in the cheese matrix, and thus the significantly higher CY (Table 1). 

### 3.5. Microstructures of Rennet Gels

**Figure 4:**
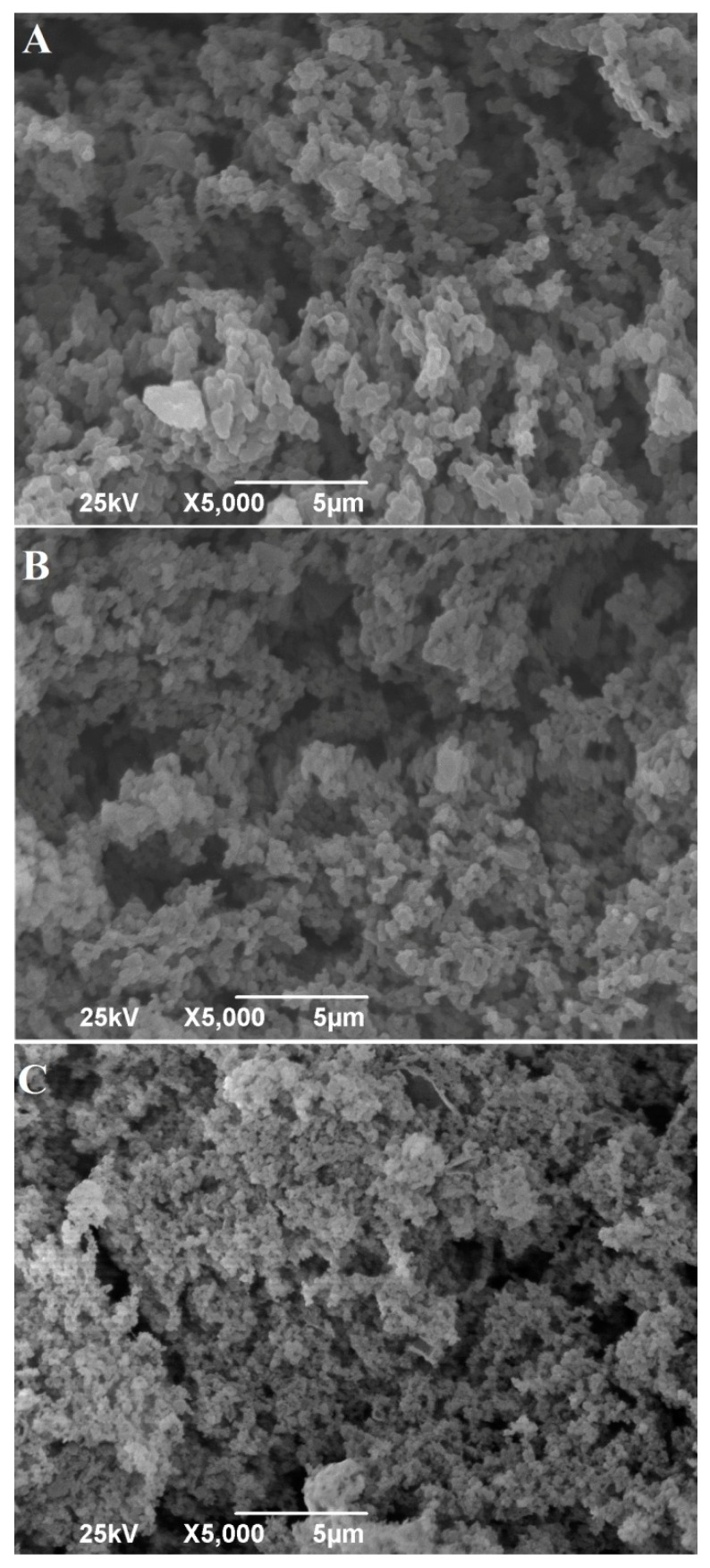
Microstructures of rennet gels from goat milk heated to 65 °C/30 min (**A**), 80 °C/5 min (**B**) and 90 °C/5 min (**C**).

The microstructures of rennet gel obtained from milk heated to 90 °C/5 min were notably different compared to the other two samples (Gc and G8) (Figure 4).

While in Gc and G8, the protein matrix was composed from large regular spheres of paracasein micelles, large void spaces and thick protein cross links, the G9 matrix was characterized by a dense, compact microstructure, with smaller paracasein micelles and void spaces in a form of cracks, indicating weak cross links. In contrast, when cow milk is heated to 80 °C and 90 °C/5 min, no differences are observed in the microstructures of rennet gels—Li and Wang [47].

Even though the great majority of compositional and textural properties of cheeses made from goat milk heated to 80 and 90 °C/5 min do not differ significantly, they differed from cheeses from commonly pasteurized milk [16].

The crucial difference between 80 °C and 90 °C/5 min treatment of goat milk is the formation of covalent aggregates between whey proteins and casein fractions in the case of higher-heat treatment [48]. By being integrated in goat milk casein micelle, as was mentioned before, such aggregates change both micelles and the way they interact during rennet coagulation. It remains to be investigated why such a change does not appear in highly pasteurized cow milk.

## 4. Conclusions

It could be concluded that heat treatment of goat milk prior to rennet coagulation affects significantly rennet coagulation, curd yield and gel microstructure. The higher the heating temperature of milk treatment, the lower the aggregation rate and curd firmness, but no significant difference in coagulation time between the treatments applied. The higher the heat treatment, the slower the coagulation and the lesser the elastic nature of gels, but the final gels were less prone to rearrangement. Storage modulus G’ at the point of gel cutting was significantly lower for G8 and G9 compared to the control sample Gc. Further investigations employing instrumental techniques are needed in order to accurately the predict cutting window for highly pasteurized goat milk. High heat treatments of goat milk caused significant increases in both laboratory curd yield (LCY) and curd yield (CY). However, LCY of G9 treatment was unreasonably high compared with CY, suggesting that in this particular case, laboratory curd yield determination is not the proper method for yield prediction. 

The microstructure of G9 was notably different compared to Gc and G8, showing that formation of covalently bonded aggregates between whey proteins and casein fractions changed paracasein micelles and the way they interact during rennet coagulation. According to literature data, when cow milk is pretreated by the same heat treatment, rennet gels do not exhibit such a difference. It remains to be investigated why such different performances occur for the two milk types. The findings of this study might serve as the bases for the development of different cheese types produced from high-heat-treated goat milk. 

## Figures and Tables

**Table 1:** Laboratory yield (LCY), yield (CY) and yield efficiency (CYE) of curd made from caprine milk heated at 65 °C/30 min, 80 °C/5 min and 90 °C/5 min *.

Heat Treatment	65 °C/30 min	80 °C/5 min	90 °C/5 min
LCY (%)	17.1 ± 1.17 ^a^	22.1±3.59 ^b^	27.7 ± 1.84 ^c^
CY (%)	10.7 ± 0.59 ^a^	14.2±1.08 ^b^	15.4 ± 0.60 ^b^
CYE (%)	62.2 ± 1.45 ^a^	65.1±6.56 ^a^	55.6 ± 3.12 ^b^

* Results in the table are the mean values of three replicates ± standard deviation. ^a,b,c^ Values within the row sharing the same superscript letter are not statistically different from one another (*p* > 0.05).
